# Pyrroloquinoline quinone ameliorates diabetic cardiomyopathy by inhibiting the pyroptosis signaling pathway in C57BL/6 mice and AC16 cells

**DOI:** 10.1007/s00394-021-02768-w

**Published:** 2022-01-08

**Authors:** Xue-feng Qu, Bing-zhong Zhai, Wen-li Hu, Min-han Lou, Yi-hao Chen, Yi-feng Liu, Jian-guo Chen, Song Mei, Zhen-qiang You, Zhen Liu, Li-jing Zhang, Yong-hui Zhang, Yin Wang

**Affiliations:** 1 Institute of Food Science and Engineering, Hangzhou Medical College, Tianmushan Road 182th, Hangzhou, 310013 Zhejiang People’s Republic of China; 2 Department of Basic Medical Science, Chongqing Three Gorges Medical College, Tianxing Road 366th, Chongqing, 404120 People’s Republic of China

**Keywords:** Diabetic cardiomyopathies, Inflammation, NF-kappa B, Pyroptosis, Pyrroloquinoline quinone

## Abstract

**Purpose:**

Diabetic cardiomyopathy (DCM), a common complication of diabetes mellitus and is characterized by myocardial hypertrophy and myocardial fibrosis. Pyrroloquinoline quinone (PQQ), a natural nutrient, exerts strong protection against various myocardial diseases. Pyroptosis, a type of inflammation-related programmed cell death, is vital to the development of DCM. However, the protective effects of PQQ against DCM and the associated mechanisms are not clear. This study aimed to investigate whether PQQ protected against DCM and to determine the underlying molecular mechanism.

**Methods:**

Diabetes was induced in mice by intraperitoneal injection of streptozotocin, after which the mice were administered PQQ orally (10, 20, or 40 mg/kg body weight/day) for 12 weeks. AC16 human myocardial cells were divided into the following groups and treated accordingly: control (5.5 mmol/L glucose), high glucose (35 mmol/L glucose), and HG + PQQ groups (1 and 10 nmol/L PQQ). Cells were treated for 24 h.

**Results:**

PQQ reduced myocardial hypertrophy and the area of myocardial fibrosis, which was accompanied by an increase in antioxidant function and a decrease in inflammatory cytokine levels. Moreover, myocardial hypertrophy—(ANP and BNP), myocardial fibrosis—(collagen I and TGF-*β*1), and pyroptosis-related protein levels decreased in the PQQ treatment groups. Furthermore, PQQ abolished mitochondrial dysfunction and the activation of NF-*κ*B/I*κ*B, and decreased NLRP3 inflammation-mediated pyroptosis in AC16 cells under high-glucose conditions.

**Conclusion:**

PQQ improved DCM in diabetic mice by inhibiting NF-*κ*B/NLRP3 inflammasome-mediated cell pyroptosis. Long-term dietary supplementation with PQQ may be greatly beneficial for the treatment of DCM.

**Graphical abstract:**

Diagram of the underlying mechanism of the effects of PQQ on DCM. PQQ inhibits ROS generation and NF-*κ*B activation, which stimulates activation of the NLRP3 inflammasome and regulates the expression of caspase-1, IL-1*β*, and IL-18. The up-regulated inflammatory cytokines trigger myocardial hypertrophy and cardiac fibrosis and promote the pathological process of DCM.

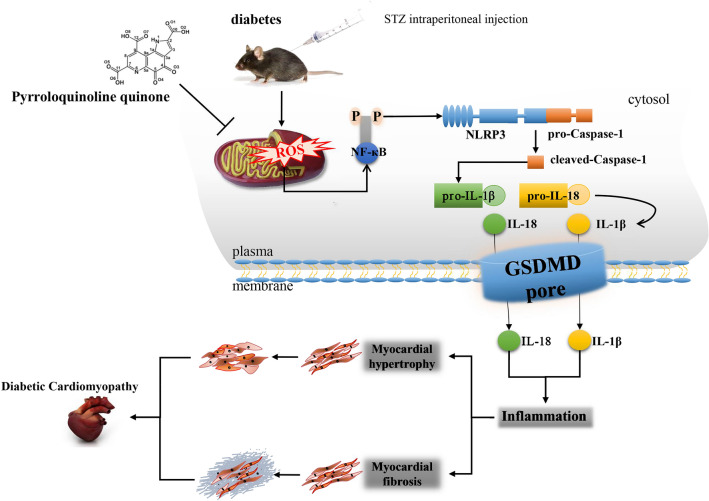

## Introduction

Diabetic cardiomyopathy (DCM) is a major cardiovascular complication of diabetes mellitus and is the leading cause of mortality in diabetic patients [1]. Structural and functional abnormalities are the main features of DCM, which is characterized by myocardial hypertrophy, cardiac fibrosis, and cardiac dysfunction [2]. The pathogenesis of DCM includes mitochondrial dysfunction, inflammation, extracellular matrix deposition, and cell death [3]. The inflammatory response has been shown to play an important role in the pathophysiology of DCM [4, 5]. Bioactive substances in food have important protective effects against cardiac diseases via anti-inflammatory and anti-oxidative effects. The use of appropriate bioactive substances combined with clinical treatment may be an effective method for DCM management.

Pyrroloquinoline quinone (PQQ), a natural bioactive substance, is a water-soluble redox coenzyme that is considered to be a new B vitamin [6]. PQQ is distributed widely in multiple dietary sources and exhibits potential health benefits, such as anti-inflammatory [7], anti-oxidative [8], anti-diabetic [9], and cardioprotective effects [10]. Several studies have demonstrated that PQQ deficiency leads to immune dysfunction, growth impairment, and abnormal reproductive performance [11]. Mammals do not synthesize PQQ and, therefore, dietary supplementation with PQQ is essential for maintaining human health. Some studies have shown that PQQ protects the heart against oxidative stress injury [12], myocardial ischemia/reperfusion injury [13], hypertrophy [14], and heart failure [15]. However, the protective effect of PQQ against DCM and the associated mechanism are not clear.

Pyroptosis, which is characterized by the activation of the Nod-like receptor family pyrin domain-containing 3 (NLRP3) inflammasome, is a type of programmed cell death associated with inflammation [10]. Recent studies have demonstrated that pyroptosis is associated with DCM [16]**.** In the diabetic state, the NLRP3 inflammasome is assembled, leading to the cleavage of caspase-1. Cleaved caspase-1 is able to convert pro-interleukin-1*β* (pro-IL-1*β*) and pro-interleukin-18 (pro-IL-18) into mature IL-1*β* and IL-18, respectively [17, 18]. In addition, several studies have reported that NF-*κ*B participates in the activation of the NLRP3 inflammasome, which triggers the transcription of NLRP3, IL-1*β*, and IL-18 [19, 20]. Luo et al.[21] found that silencing NLRP3 attenuates pyroptosis, cardiac fibrosis, cardiac inflammation, and cardiac dysfunction. In another study, the lncRNA-Kcnq1ot1/miR-214/NLRP3/caspase-1 signaling pathway was found to be a vital mechanism for the development of DCM [18]. Thus, targeting pyroptosis signaling molecules, such as NLRP3, caspase-1, IL-1*β*, and IL-18, has potential for the prevention and treatment of DCM.

In this study, we demonstrated for the first time that PQQ significantly alleviated myocardial damage, including myocardial fibrosis and myocardial hypertrophy, in diabetic mice. Further, PQQ was found to inhibit the activation of the NF-*κ*B/pyroptosis signaling pathway in diabetic mice and high glucose-treated AC16 cells.

## Materials and methods

### Animals and establishment of the DCM model

PQQ, with a purity > 98%, was provided by Zhucheng HaoTian Pharm Co., Ltd. (Shandong, China). Male C57BL/6 mice (8 weeks old) were obtained from the Animal Experiment Center of Zhejiang Academy of Medicine Sciences (Hangzhou, China). All mice were housed at 22 ± 1 ℃ and 55 ± 5% humidity, with ad libitum access to food and water. The mice were randomly divided into the following five groups (*n* = 10 for each group): controls (Ctl), diabetic cardiomyopathy (DCM), diabetic mice treated with a low dose of PQQ (DCM + PQQL), diabetic mice treated with a medium dose of PQQ (DCM + PQQM), and diabetic mice treated with a high dose of PQQ (DCM + PQQH). Type 1 diabetes was induced by intraperitoneal injection (i.p.) of 50 mg/kg/day streptozotocin (STZ; Sigma, St. Louis, MO, USA) for 5 days. Five days after STZ injection, fasting blood glucose concentrations were measured using a contour glucose meter (Roche, Basel, Switzerland), and mice with glucose concentrations ≥ 16.7 mmol/L were used in the experiments. Subsequently, some diabetic mice were treated with PQQ orally (10, 20, or 40 mg/kg body weight/day). All mice were fed for 12 weeks and were then anesthetized with 3% pentobarbital sodium.

### Cell culture and treatment

AC16 human myocardial cells were purchased from Otwo Biotech (HTX2571, Shenzhen, China) and were cultured in Dulbecco’s modified Eagle’s medium (DMEM; Gibco, Grand Island, NY, USA) with 10% fetal bovine serum (Biological Industries, Haemek, Israel) and 1% penicillin–streptomycin (TBD, Tianjin China), in an incubator at 37 °C and 5% CO_2_. The cells were then divided into the following groups and treated accordingly: control (5.5 mmol/L glucose), high glucose (HG, 35 mmol/L glucose), and HG + PQQ groups (1 and 10 nmol/L PQQ). Cells were treated for 24 h.

### Cell viability assay

An MTT assay kit (Keygen Biotech, Jiangsu, China) was used to determine the protective effect of different concentrations of PQQ against HG-induced cell damage, according to the manufacturer’s instructions. After AC16 cells were passaged stably, they were seeded into 96-well plates at a density 1 × 10^4^ cells/well. PQQ was added to the 96-well plate at increasing concentrations (1, 10, 100, 1000, 5000, 10,000, and 100,000 nmol/L). After 24 h, the cells were incubated with 50 μL of 1 × MTT for 4 h and then, 150 μL of DMSO was added to each well. Absorbance was measured at 490 nm.

### Hematoxylin and eosin and Masson’s trichrome staining

Murine heart tissues were fixed with 4% paraformaldehyde and embedded in paraffin. They were then cut into 5 μm sections and stained using hematoxylin and eosin (H&E) and Masson’s trichrome protocols. The sections of the left ventricle were observed under an optical microscope (Olympus Corporation, Tokyo, Japan) at 400×, then the morphology of cardiomyocytes determined and collagen deposition in the interstitial and perivascular regions measured. The cardiomyocyte area and fibrotic area were determined using Image-Pro Plus imaging software (Media Cybernetics, Rockville, MD, USA).

### RNA isolation and real-time RT-PCR

Total RNA was extracted from the left ventricle of mouse hearts using TRIzol (Tiangen Biotech, Beijing, China). A NanoDrop spectrophotometer (Wilmington, DE, USA) was used to determine RNA concentration. cDNA was synthesized using a reverse transcription kit (Life Technologies, Grand Island, NY, USA), and the cDNA was amplified and quantified using an ABI 7500 Fast real-time PCR system (Applied Biosystems, Foster City, CA, USA) with SYBR Green (Life Technologies, Grand Island, NY, USA). The reaction conditions were 95 ℃ for 5 min, followed by 40 cycles of 95 ℃ for 15 s, 60 ℃ for 30 s, and 72 ℃ for 45 s. mRNA expression levels were normalized to GAPDH levels. Relative mRNA expression levels were determined using the 2^−△△Ct^ method. The primers sequences are listed in Table 1.

**Table 1:** Primer sequences used in RT-PCR assays

Gene	Forward primer (5′–3′)	Reverse primer (5′–3′)
ANP	AACCTGCTAGACCACCTGGA	TGCTTTTCAAGAGGGCAGAT
BNP	GAAGGACCAAGGCCTCACAAA	AACTTCAGTGCGTTACAGCC
*β*-MHC	CCAGAAGCCTCGAAATGTC	CTTTCTTTGCCTTGCCTTTGC
Col I	CAATGGCACGGCTGTGTGCG	CACTCGCCCTCCCGTCTTTGG
Col III	TGGCACAGCAGTCCAACGTA	AAGGACAGATCCTGAGTCACAGACA
TGF-*β*1	GTGTGGAGCAACATGTGGAACTCTA	TTGGTTCAGCCACTGCCGTA
CTGF	AGAACTGTGTACGGAGCGTG	GTGCACCATCTTTGGCAGTG
caspase-1	ACACGTCTTGCCCTCATTATCT	ATAACCTTGGGCTTGTCTTTCA
NLRP3	GTGGAGATCCTAGGTTTCTCTG	CAGGATCTCATTCTCTTGGATC
GAPDH	AAGAAGGTGGTGAAGCAGGC	TCCACCACCCTGTTGCTGTA

### Western blotting analysis

Total protein was extracted using RIPA lysis buffer containing 1% protease inhibitor, and the concentration of the extracted protein was measured using a BCA kit. Equal amounts of protein were separated by 8–15% SDS-PAGE and transferred to nitrocellulose (NC) membranes. The NC membranes were blocked with 5% bovine serum albumin for 2 h at room temperature and then incubated with primary antibodies overnight at 4 ℃. The primary antibodies against different target proteins were listed as follows: NLRP3 (BA3677, Boster Biological Technology, Wuhan, China), caspase-1 (24232s, Cell Signaling Technology, Danvers, MA, U.S.A.), cleaved caspase-1 (4199s, Cell Signaling Technology, Danvers, MA, U.S.A.), IL-1*β* (12242s, Cell Signaling Technology, Danvers, MA, U.S.A.), IL-18 (54943s, Cell Signaling Technology, Danvers, MA, U.S.A.), NF-*κ*B p65 (8242s, Cell Signaling Technology, Danvers, MA, U.S.A.), phospho-NF-*κ*B p65 (3033s, Cell Signaling Technology, Danvers, MA, U.S.A.), I*κ*B*α* (9242s, Cell Signaling Technology, Danvers, MA, U.S.A.), phospho-I*κ*B*α* 9246s, Cell Signaling Technology, Danvers, MA, U.S.A.), collagen III (ab7778, Abcam, Cambridge, MA, U.S.A.), collagen I (14695-1-AP, Proteintech, Wuhan, China), TGF-*β*1 (ab9758, Abcam, Cambridge, MA, U.S.A.), *β*-actin (BK7018, Bioker, Hangzhou, China), and GAPDH (BK7021, Bioker, Hangzhou, China). NC membranes were then incubated with fluorescently labeled secondary antibodies (LI-COR Biosciences, Lincoln, NE, USA) for 1 h at room temperature in the dark. GAPDH and *β*-actin were used for normalization. The results were captured and analyzed using an Odyssey Imaging System (LI-COR Biosciences).

### ELISA analysis

ELISAs were performed to determine the levels of IL-1*β*, IL-6, and TNF-*α* in heart tissue using commercially available kits (QuantiCyto, Shanghai, China) according to the manufacturer’s instructions. Total protein was extracted from heart tissues and the concentration of protein was determined using a BCA kit.

### Assessment of oxidative stress

Heart tissue (0.3–0.4 g) was washed with cold saline to remove blood. A 10% tissue homogenate was prepared by completely grinding the heart tissue in nine volumes (w/v) of ice-cold saline using a grinding rod. The homogenate was then centrifuged at 3000×*g* for 15 min at 4 ℃ to separate the cell debris. All procedures were performed in accordance with the corresponding kit manufacturer’s protocol. Lactate dehydrogenase (LDH), creatine kinase MB (CK-MB), superoxide dismutase (SOD), glutathione peroxidase (GSH-px), and catalase (CAT) activity were measured using commercially available assay kits (Jiancheng, Nanjing, China). Malondialdehyde (MDA) concentrations were detected using a commercially available MDA assay kit (Jiancheng, Nanjing, China).

### Detection of reactive oxygen species

AC16 cells were seeded into 6-well plates and treated with 35 mmol/L glucose and PQQ for 24 h. To measure intracellular reactive oxygen species (ROS) concentrations, the cells were incubated with 10 μmol/L 2ʹ,7ʹ-dichlorofluorescein diacetate (DCFH-DA; Abcam, Cambridge, MA, USA) for 45 min at 37 ℃. The cells were then washed three times with serum-free medium, and DCFH-DA fluorescence was observed using a fluorescence microscope (Olympus Corporation; magnification 400×). Fluorescence intensities were quantified using Image-Pro Plus (version 5.0; Media Cybernetics, Inc., Rockville, MD, USA).

### Mitochondrial membrane potential detection

A JC-1 mitochondrial membrane potential assay kit (MedChemExpress, Shanghai, China) was used to detect changes in the mitochondrial membrane potential in AC16 cells treated with HG and PQQ. AC16 cells were cultured in six-well plates at a density of 5 × 10^5^ cells/well, and incubated with 10 μL of JC-1 (200 μmol/L) for 20 min at 37 ℃. The images were captured using a fluorescence microscope (Olympus Corporation; magnification × 400).

### Statistical analysis

Results are presented as mean ± standard deviation (SD) of at least three independent experiments. Multiple groups were analyzed using a one-way ANOVA. Differences were considered statistically significant if *P* < 0.05. Statistical analyses were performed using SPSS software (version 22.0; IBM, Armonk, NY, USA).

## Results

### Effects of PQQ on fasting blood glucose concentrations and heart weight in diabetic mice

Diabetic mice showed increased fasting blood glucose concentrations (22.19 ± 1.37 vs. 6.73 ± 0.17 mmol/L) and decreased body weight (23.16 ± 0.76 g vs. 26.88 ± 0.43 g) compared with mice in the control group. Treatment with 40 mg/kg/day PQQ significantly reduced the blood glucose concentrations of diabetic mice, but had no effect on body weight. The heart weight/body weight ratio significantly increased in the diabetic group, and this was ameliorated by treatment with 40 mg/kg/day PQQ (Table 2).

**Table 2:** Effects of PQQ on blood glucose concentrations and heart weight in mice with STZ-induced diabetes

Groups	Ctl	DCM^c^	DCM + PQQL^d^	DCM + PQQM^e^	DCM + PQQH^f^
	*n* = 10	*n* = 10	*n* = 10	*n* = 10	*n* = 10
Blood glucose (mmol/L)	6.73 ± 0.55	22.19 ± 4.34***	21.45 ± 4.33	19.66 ± 3.61	17.83 ± 2.28^#^
BW ^a^(g)	26.88 ± 1.37	23.16 ± 2.39***	22.77 ± 1.38	23.38 ± 2.61	23.81 ± 1.18
HW/BW ^b^(mg/g)	4.06 ± 0.31	4.77 ± 0.54*	4.18 ± 0.61	4.13 ± 0.38	3.70 ± 0.72^###^

Data are presented as the mean ± SD

*P < 0.05

***P < 0.001 vs. Ctl

^#^*P* < 0.05

^##^*P* < 0.01

^###^*P* < 0.001 vs. DCM

^a^*BW* body weight

^b^*HW/BW* heart weight/body weight ratio

^c^*DCM* diabetic cardiomyopathy group

^d^*DCM + PQQL* diabetic mice treated with 10 mg/kg BW/day PQQ

^e^*DCM + PQQM* diabetic mice treated with 20 mg/kg BW/day PQQ

^f^*DCM + PQQH* diabetic mice treated with 40 mg/kg BW/day PQQ

### PQQ ameliorated diabetes-induced myocardial hypertrophy

**Fig. 1:**
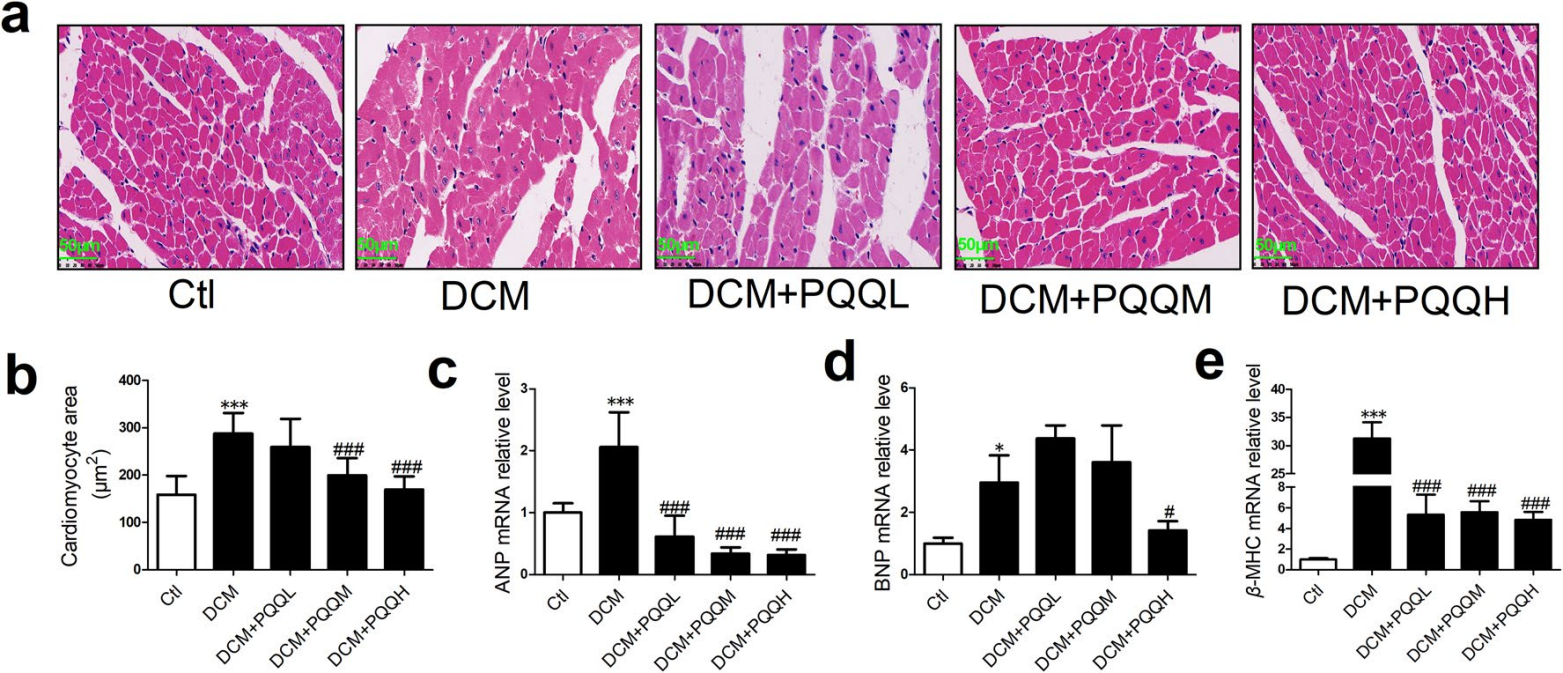
PQQ ameliorated diabetes-induced myocardial hypertrophy. **a** Representative H&E staining in the study groups. Chromatin and nucleic acids are stained blue and the cytoplasm and extracellular matrix are stained red; magnification, × 400; *n* = 5 per group. **b** Comparison of cardiomyocyte area in the mice from each group; *n* = 5. Relative mRNA expression levels of **c** ANP, **d** BNP, and **e** *β*-MHC were measured by real-time RT-PCR; *n* = 4 per group. Data are presented as mean ± SD, *P < 0.05, ***P < 0.001 vs. Ctl, ^#^*P* < 0.05, ^###^*P* < 0.001 vs. DCM. *DCM* diabetic cardiomyopathy group, *DCM + PQQL* diabetic mice treated with 10 mg/kg BW/day PQQ, *DCM + PQQM* diabetic mice treated with 20 mg/kg BW/day PQQ, *DCM + PQQH* diabetic mice treated with 40 mg/kg BW/day PQQ

The effects of PQQ on myocardial hypertrophy were determined in diabetic mice. Cardiomyocyte area was significantly increased in diabetic mice compared with control mice, and this was attenuated by PQQ treatment at 20 or 40 mg/kg/day (Fig. 1a, b). The levels of markers of myocardial hypertrophy, including atrial natriuretic peptide (ANP), brain natriuretic peptide (BNP), and myosin heavy chain beta (*β*-MHC), were increased in the DCM group. However, PQQ treatment ameliorated this increase in ANP, BNP, and *β*-MHC mRNA levels (Fig. 1c–e). Thus, PQQ had a protective effect against myocardial hypertrophy in DCM.

### PQQ ameliorated diabetes-induced cardiac fibrosis

**Fig. 2:**
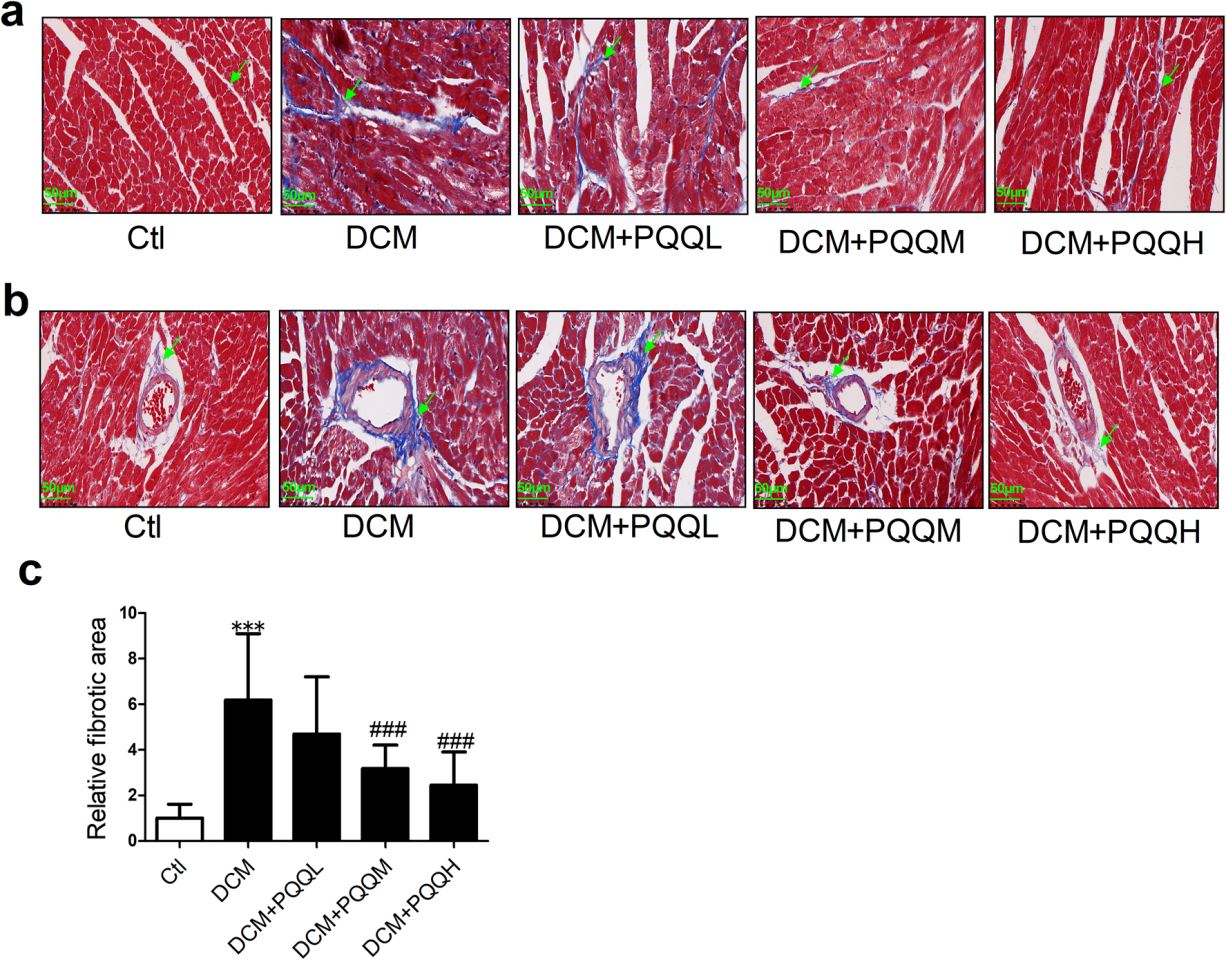
PQQ ameliorated diabetes-induced cardiac fibrosis. **a** Representative Masson’s trichrome staining of interstitial fibrosis in the study groups; magnification, × 400; *n* = 5 per group. **b** Representative Masson’s trichrome staining of perivascular fibrosis in the study groups; magnification, × 400; *n* = 5. Collagen is stained blue and the myocardium is stained red. **c** Analysis of cardiac fibrosis area; *n* = 5 per group. Data are presented as mean ± SD, ***P < 0.001 vs. Ctl, ^###^*P* < 0.001 vs. DCM. DCM. *DCM* diabetic cardiomyopathy group, *DCM + PQQL* diabetic mice treated with 10 mg/kg BW/day PQQ, *DCM + PQQM* diabetic mice treated with 20 mg/kg BW/day PQQ, *DCM + PQQH* diabetic mice treated with 40 mg/kg BW/day PQQ

**Fig. 3:**
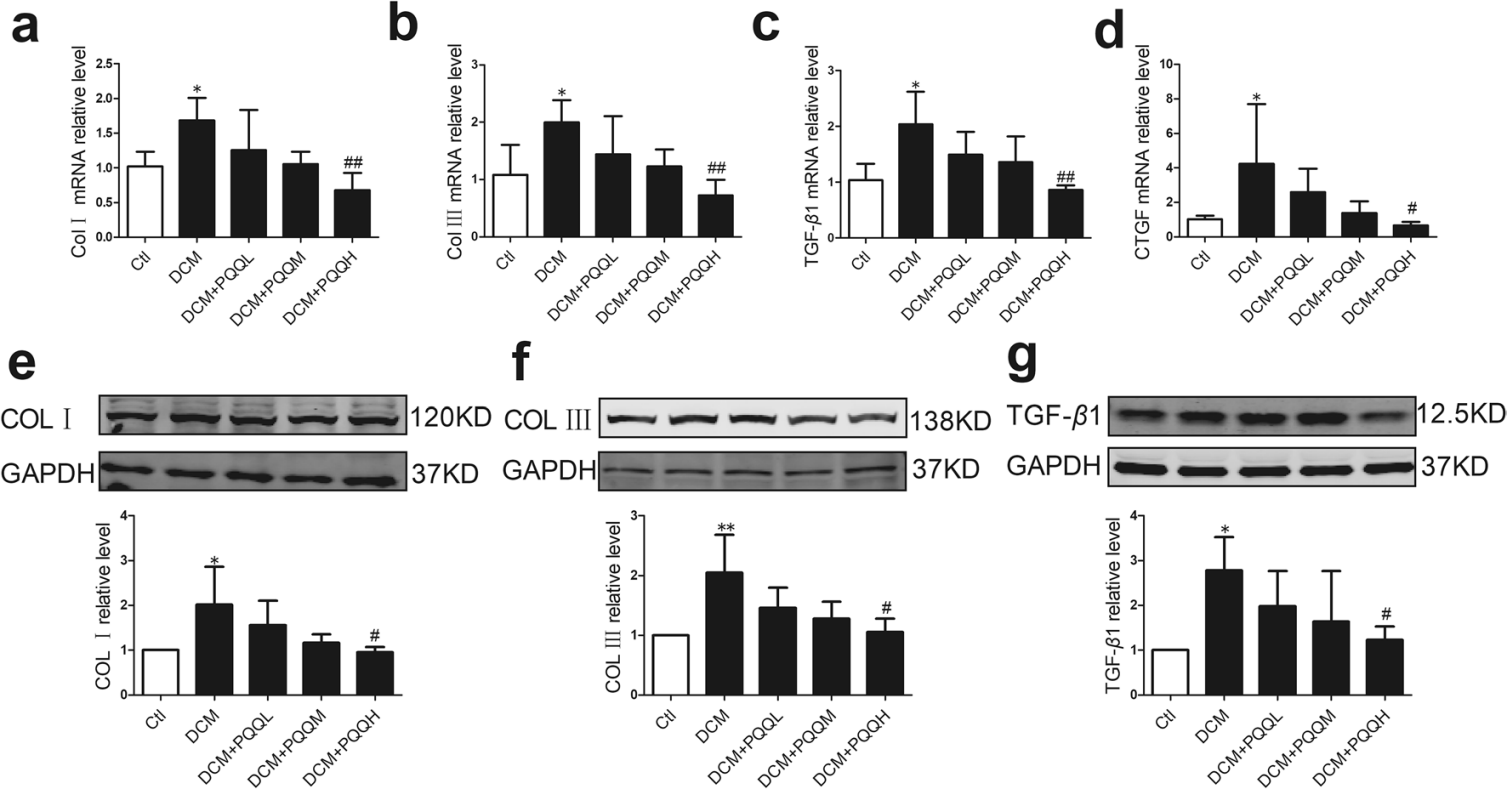
PQQ ameliorated cardiac fibrosis-related mRNA and protein levels. The relative mRNA expression levels of **a** Col I (*n* = 5), **b** Col III (*n* = 5), **c** TGF-*β*1 (*n* = 4), and **d** CTGF (*n* = 5) were measured by real-time RT-PCR. Protein expression levels of **e** COL I, **f** COL III, and **g** TGF-*β*1 were determined by western blotting analysis in the study groups, *n* = 4 per group. Data are presented as mean ± SD, *P < 0.05, **P < 0.01 vs. Ctl, ^#^*P* < 0.05, ^##^*P* < 0.01 vs. DCM. DCM. *DCM* diabetic cardiomyopathy group, *DCM + PQQL* diabetic mice treated with 10 mg/kg BW/day PQQ, *DCM + PQQM* diabetic mice treated with 20 mg/kg BW/day PQQ, *DCM + PQQH* diabetic mice treated with 40 mg/kg BW/day PQQ

As shown in Fig. 2, collagen deposition in the myocardial interstitial and perivascular regions was significantly up-regulated in diabetic mice. However, treatment with 20 or 40 mg/kg/day PQQ decreased the area of fibrosis induced by diabetes. To further determine the effects of PQQ on cardiac fibrosis, we examined the levels of fibrosis-related mRNAs, including collagen I (Col I), collagen III (Col III), transforming growth factor-*β*1 (TGF-*β*1), and connective tissue growth factor (CTGF). As shown in Fig. 3a–d, the mRNA levels of Col I, Col III, TGF-*β*1, and CTGF were all increased in STZ-treated mice, but PQQ treatment recovered the levels of these mRNAs. Furthermore, PQQ treatment significantly inhibited the protein expression levels of COL I, COL III, and TGF-*β*1 in diabetic mice (Fig. 3e–g). These results demonstrated that PQQ had a protective effect against cardiac fibrosis in DCM.

### PQQ ameliorated diabetes-induced inflammation

**Fig. 4:**
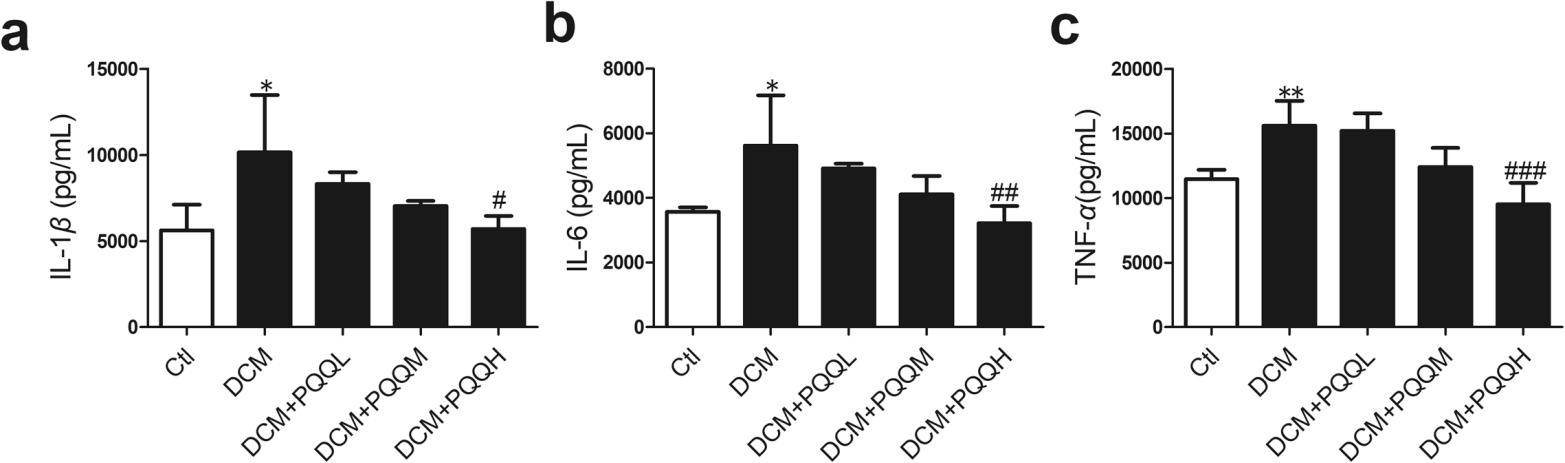
PQQ ameliorated diabetes-induced inflammation. Levels of the inflammatory cytokines **a** IL-1*β*, **b** IL-6, and **c** TNF-*α* were measured by ELISA; *n* = 4 per group. Data are presented as mean ± SD, *P < 0.05, **P < 0.01 vs. Ctl, ^#^*P* < 0.05, ^##^*P* < 0.01, ^###^*P* < 0.001 vs. DCM. DCM. *DCM* diabetic cardiomyopathy group, *DCM + PQQL* diabetic mice treated with 10 mg/kg BW/day PQQ, *DCM + PQQM* diabetic mice treated with 20 mg/kg BW/day PQQ, *DCM + PQQH* diabetic mice treated with 40 mg/kg BW/day PQQ

To determine the protective role of PQQ against diabetes-induced inflammation, the IL-1*β*, IL-6, and TNF-*α* levels were assessed in heart tissue using ELISAs. The induction of diabetes increased the levels of IL-1*β*, IL-6, and TNF-*α* in the DCM model, and these effects were inhibited by PQQ (Fig. 4). The release of proinflammatory factors is associated with NLRP3 inflammasome activation. These results demonstrated that PQQ had anti-inflammatory actions in DCM.

### PQQ protected the heart from diabetes-induced oxidative stress

**Fig. 5:**
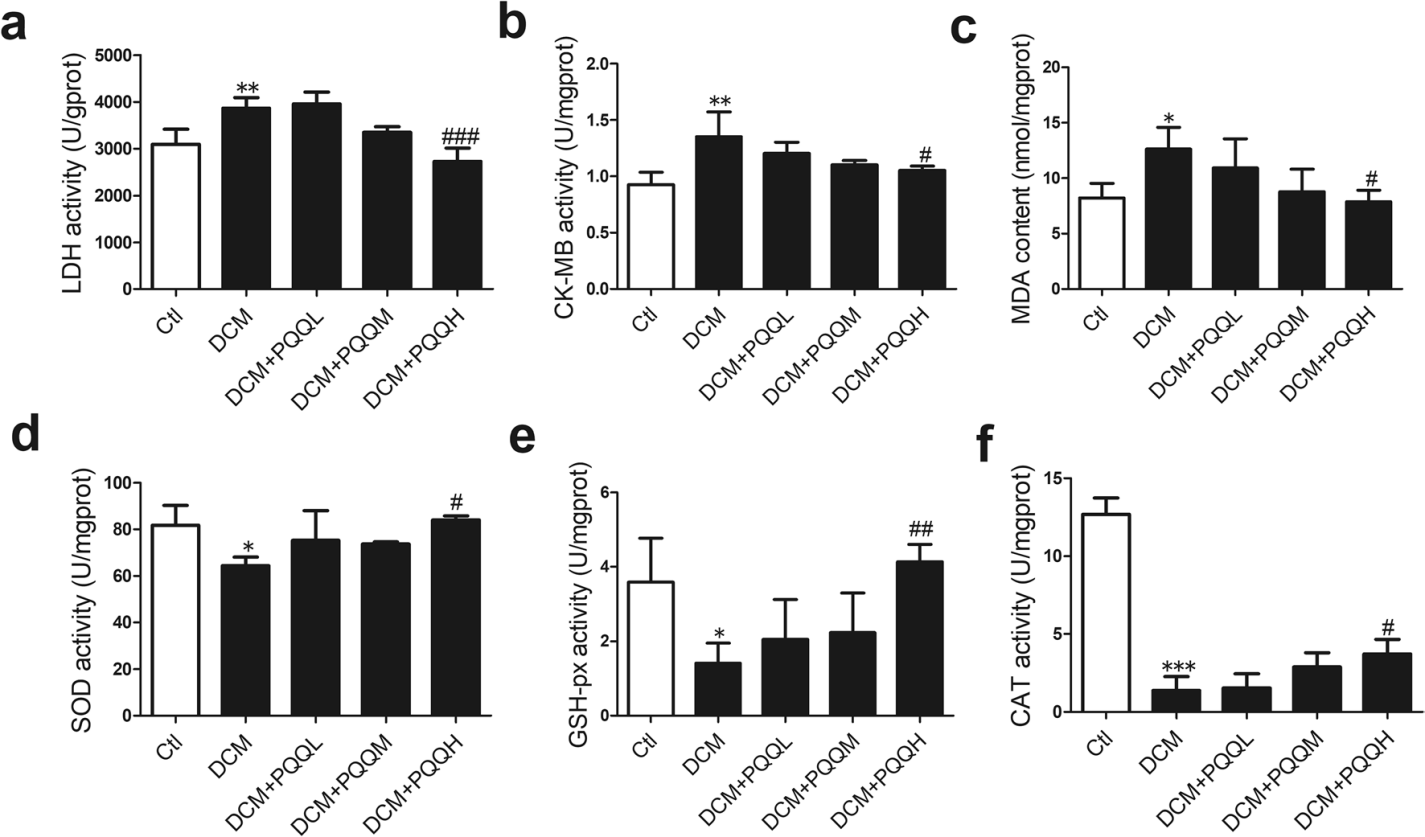
PQQ protected the heart from diabetes-induced oxidative stress. The activity or quantity of the myocardial damage-related indicators, **a** LDH and **b** CK-MB, and the oxidative stress-related indicators, **c** MDA, **d** SOD, **e** GSH-px, and **f** CAT were measured using commercial kits; *n* = 4 per group. Data are presented as mean ± SD, *P < 0.05, **P < 0.01, ***P < 0.001 vs. Ctl, ^#^*P* < 0.05, ^##^*P* < 0.01, ^###^*P* < 0.001 vs. DCM. DCM. *DCM* diabetic cardiomyopathy group, *DCM + PQQL* diabetic mice treated with 10 mg/kg BW/day PQQ, *DCM + PQQM* diabetic mice treated with 20 mg/kg BW/day PQQ, *DCM + PQQH* diabetic mice treated with 40 mg/kg BW/day PQQ

LDH and CK-MB are well-known indicators of myocardial damage. The LDH and CK-MB activities were higher in the diabetic group than the control group, but treatment with PQQ significantly ameliorated this increased activity (Fig. 5a, b). To determine whether PQQ ameliorated diabetes-induced oxidative stress, we measured the activities of MDA, SOD, GSH-px, and CAT in heart tissue. The SOD, GSH-px, and CAT activities were dramatically decreased and the MDA content was increased in the DCM group. However, these enzymatic activities were restored by PQQ treatment (Fig. 5c–f). These results showed that PQQ attenuated oxidative stress in DCM.

### Effects of PQQ on the pyroptosis signaling pathway in DCM

**Fig. 6:**
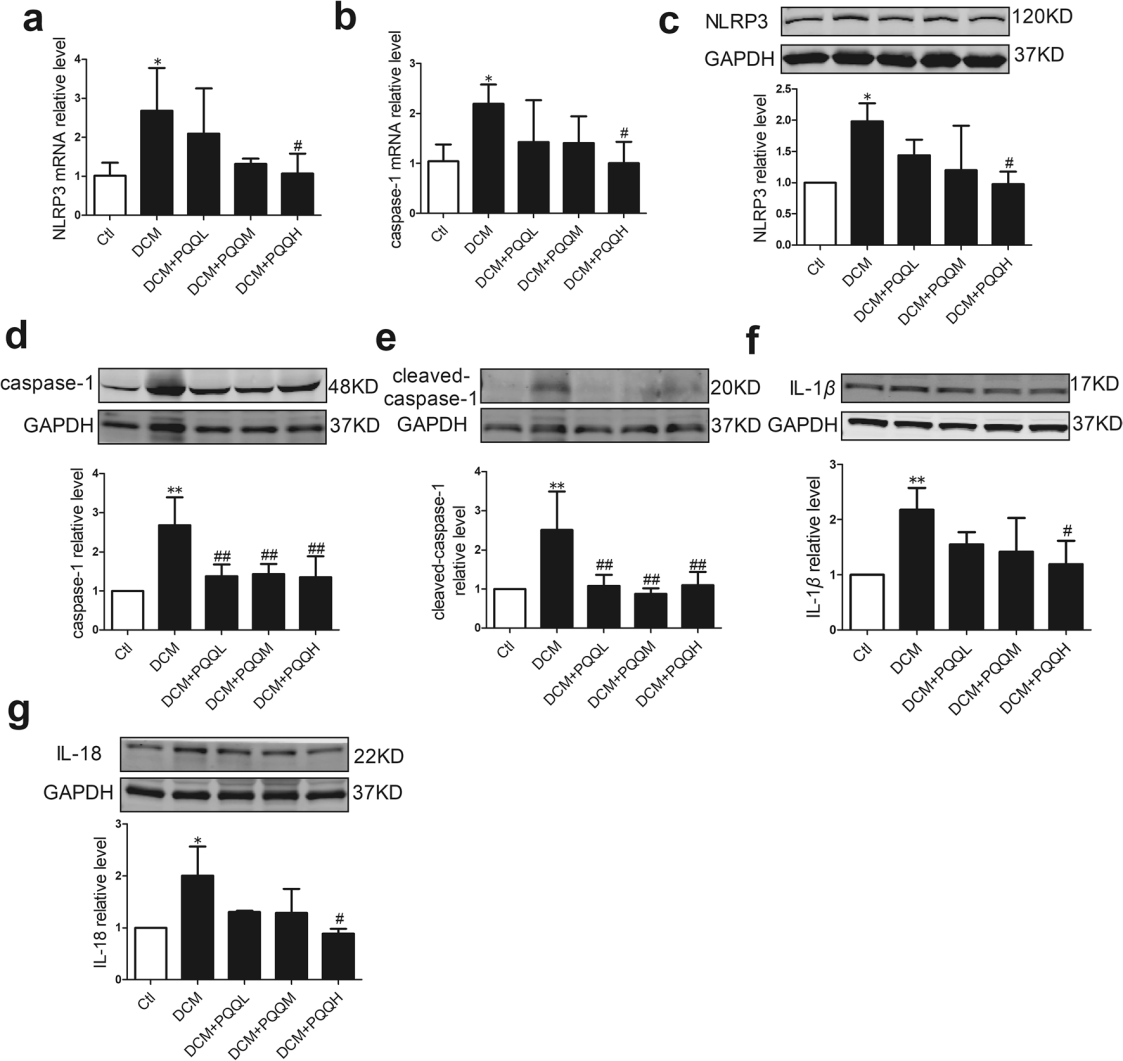
PQQ ameliorated the diabetes-induced pyroptosis of cardiomyocytes. The relative mRNA expression levels of **a** NLRP3, and **b** caspase-1 were measured by real-time RT-PCR; *n* = 5. The protein levels of **c** NLRP3 (*n* = 4), **d** caspase-1 (*n* = 4), **e** activated-caspase-1 (*n* = 4), **f** IL-1*β* (*n* = 4), and **g** IL-18 (*n* = 3) were determined by western blotting analysis. Data are presented as mean ± SD, *P < 0.05, **P < 0.001 vs. Ctl, ^#^*P* < 0.05, ^##^*P* < 0.01 vs. DCM. DCM. *DCM* diabetic cardiomyopathy group, *DCM + PQQL* diabetic mice treated with 10 mg/kg BW/day PQQ, *DCM + PQQM* diabetic mice treated with 20 mg/kg BW/day PQQ, *DCM + PQQH* diabetic mice treated with 40 mg/kg BW/day PQQ

To determine the effect of PQQ on the pyroptosis signaling pathway in this DCM model, we measured changes in NLRP3, caspase-1, IL-1*β*, and IL-18 levels. The mRNA and protein expression levels of NLRP3 and caspase-1 were up-regulated in diabetic mice, but these levels were restored by PQQ treatment (Fig. 6a–d). Moreover, the protein levels of cleaved caspase-1, IL-1*β*, and IL-18 were markedly increased in the DCM model, and these changes were reversed by PQQ treatment (Fig. 6e–g).

### The effects of PQQ on cell viability in response to HG

**Fig. 7:**
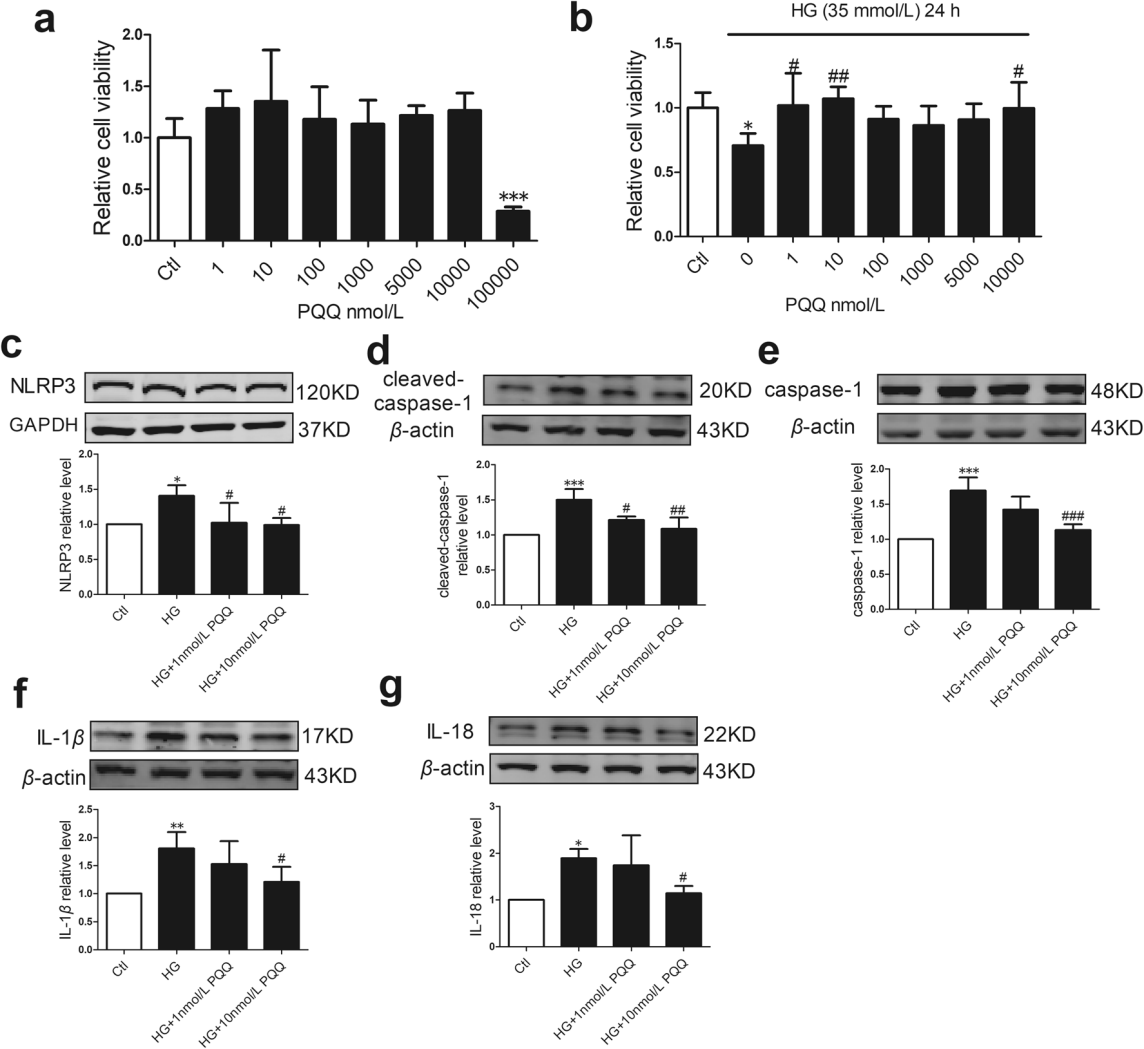
The effects of PQQ on cell viability and pyroptosis signaling in AC16 cells under HG conditions. **a** & **b** AC16 cells were treated with different concentrations of PQQ (1, 10, 100, 1,000, 5,000, 10,000, and 10,0000 nmol/L) in basic DMEM (5.5 mmol/L glucose) or HG (35 mmol/L glucose) media for 24 h. Cell viability was measured by MTT assay, *n* = 6. The protein levels of **c** NLRP3, **d** cleaved-caspase-1, **e** caspase-1, **f** IL-1*β*, and **g** IL-18 were analyzed by western blotting, *n* = 4 per group. Mean ± SD, *P < 0.05, **P < 0.01, ***P < 0.001 vs. Ctl, ^#^*P* < 0.05, ^##^*P* < 0.01, ^###^*P* < 0.001 vs. HG. *HG* high glucose; HG + 1 nmol/L PQQ, high glucose with 1 nmol/L PQQ; HG + 10 nmol/L PQQ, high glucose with 10 nmol/L PQQ

To determine the effect of different concentrations of PQQ on AC16 cells, we used an MTT assay kit to determine cell viability. AC16 cells were treated with increasing concentrations of PQQ (1, 10, 100, 1000, 5000, 10,000, and 100,000 nmol/L) for 24 h. At 100,000 nmol/L, PQQ caused significant cellular damage, whereas the other tested concentrations of PQQ had no obvious effect on AC16 cells under normal conditions (Fig. 7a). Therefore, AC16 cells were treated with 1–10,000 nmol/L PQQ under HG conditions. HG markedly inhibited cell viability compared with control cells, and PQQ protected AC16 cells from HG-induced damage (Fig. 7b).

### PQQ ameliorated pyroptosis signaling in AC16 cells under HG conditions

Based on the previous results, 1 nmol/L and 10 nmol/L were considered to be the minimum effective PQQ concentrations needed to protect against HG-induced damage. Thus, these concentrations were selected for subsequent experiments. As shown in Fig. 7c–g, the NLRP3, cleaved-caspase-1, caspase-1, IL-1*β*, and IL-18 protein levels were markedly increased in the HG group, but were decreased after treatment with 10 nmol/L PQQ. This indicated that 10 nmol/L PQQ may have a protective effect against the pyroptosis signaling pathway in AC16 cells under HG conditions.

### PQQ ameliorated NF-*κ*B activation in AC16 cells under HG conditions

**Fig. 8:**
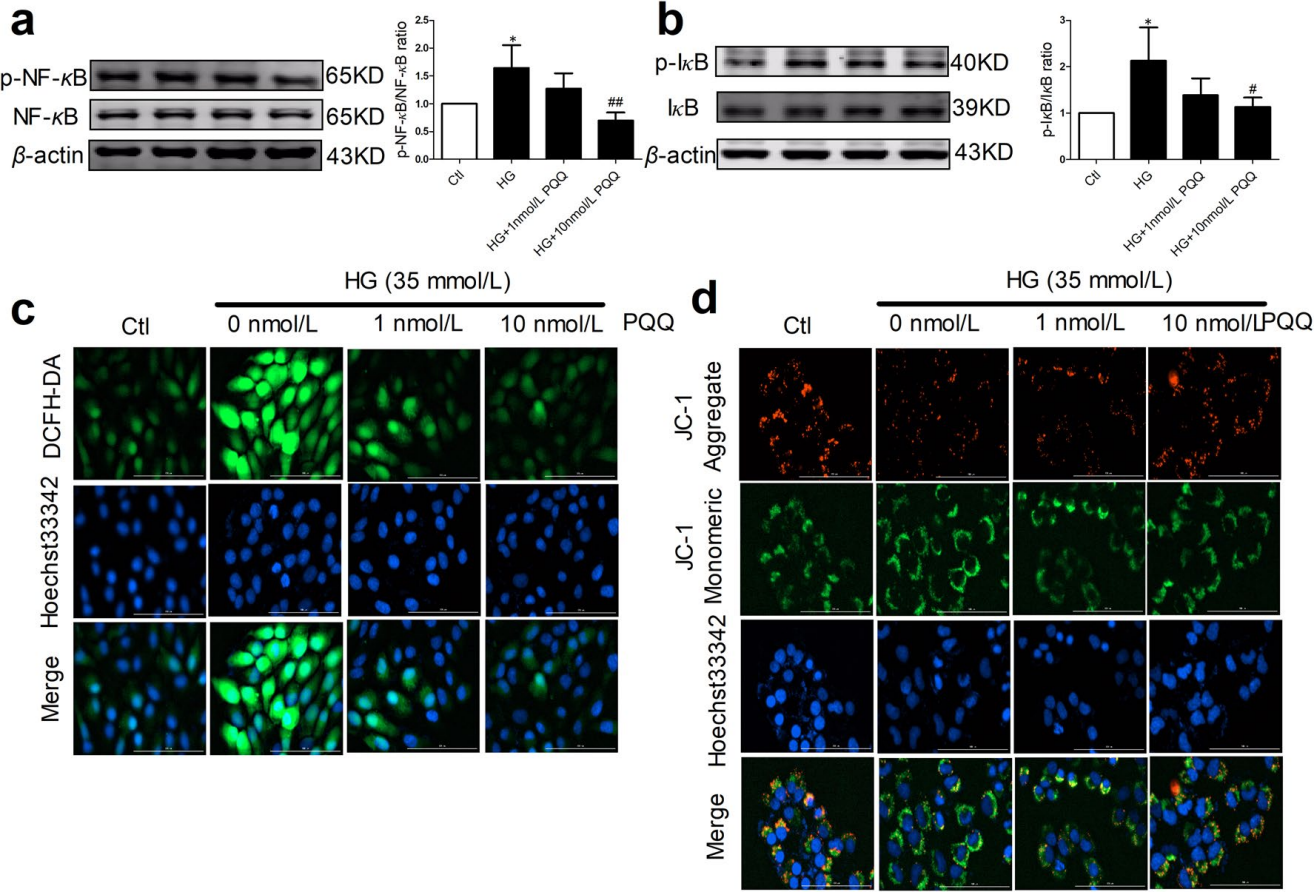
PQQ ameliorated NF-*κ*B activation and ROS generation in AC16 cells under HG conditions. **a** Protein levels of phosphorylated and total NF-*κ*B p65 were determined by western blotting analysis, *n* = 4. **b** Protein levels of phosphorylated I*κ*B and total I*κ*B were determined by western blotting analysis, *n* = 4; **c** Intracellular ROS levels were determined using a DCFH-DA assay kit, *n* = 3. **d** Mitochondrial membrane potential was measured in AC16 cells using a JC-1 mitochondrial membrane potential assay kit, *n* = 3. Mean ± SD, *P < 0.05 vs. Ctl, ^#^*P* < 0.05, ^##^*P* < 0.01 vs. HG. *HG* high glucose; HG + 1 nmol/L PQQ, high glucose with 1 nmol/L PQQ; HG + 10 nmol/L PQQ, high glucose with 10 nmol/L PQQ

The NF-*κ*B signaling pathway triggers the activation of the NLRP3 inflammasome. As shown in Fig. 8a, b, the phosphorylation levels of NF-*κ*B p65 and I*κ*B were significantly elevated after HG stimulation. However, 10 nmol/L PQQ decreased the phosphorylation levels of NF-*κ*B p65 and I*κ*B.

### PQQ ameliorated ROS generation in AC16 cells under HG conditions

To investigate the effects of PQQ on HG-induced ROS production, we measured the intracellular ROS levels and mitochondrial membrane potential. HG strongly stimulated ROS accumulation and repressed mitochondrial membrane potential in AC16 cells. However, PQQ ameliorated these effects on ROS production and of mitochondrial membrane potential (Fig. 8c, d).

## Discussion

Multiple studies have shown that myocardial hypertrophy and myocardial fibrosis are common structural abnormalities in diabetic patients, leading to cardiac stiffness and cardiac dysfunction [22]. This study, for the first time, showed that dietary supplementation with PQQ had protective effects against myocardial hypertrophy and myocardial fibrosis in diabetic mice. We showed that PQQ significantly inhibited inflammation and increased antioxidant ability. Furthermore, the cardioprotective effects of PQQ were, in part, mediated by the NF-*κ*B/NLPR3 inflammasome signaling pathway.

PQQ is the third most common redox coenzyme besides nicotinamide nucleotides and flavin nucleotides. It is widely present in vegetables and meat [23]. In humans, free PQQ is found in the heart, brain, liver, lungs, plasma, and breast milk [24], and it is necessary for maintaining the health of mammals. Dietary supplementation with PQQ has potential effects of improving the blood lipid profile, controlling blood glucose concentrations, and preventing cardiovascular diseases [25]. Devasani et al. reported that treatment with 10 or 20 mg/kg PQQ for 5 weeks improved glucose tolerance, insulin resistance, and plasma insulin concentrations, and attenuated serum concentrations of inflammatory cytokines (IL-1*β*, IL-6, and TNF-*α*) in rats fed a 10% fructose diet for 10 weeks [26]. Moreover, serum glucose concentrations have been reported to ameliorate the increase in antioxidant activity in diabetic mice receiving 20 mg/kg body weight/day PQQ (i.p.) [27]. Akagawa et al. showed that oral supplementation with PQQ (20 mg/kg day) remarkably alleviated impaired glucose tolerance in type 2 diabetic KK-*A*^y^ mice [6]. It has also been reported that 15 mg/kg PQQ (i.p.) mitigated myocardial infarct size and improved cardiac function after myocardial ischemia/reperfusion injury in rats [25]. In addition, in our previous study, oral PQQ administration (10, 20, 40 mg/kg day) for 4 weeks, was found to 40 mg/kg PQQ could significantly improve the oxidative injury of nerve cells and increase the learning and memory ability of aging rats [28]. However, the cardioprotective role of PQQ in diabetic mice has not previously been investigated.

The markers of myocardial hypertrophy, ANP, BNP, and *β*-MHC, were up-regulated in the heart tissue of diabetic mice. We found that oral administration of PQQ decreased myocardial hypertrophy-related mRNA levels and myocardial cell area. Other structural changes observed in DCM are myocardial interstitial fibrosis and perivascular fibrosis, which are characterized by increased COL I and COL III levels, resulting in impaired cardiac contractile function and diastolic function [29]. We found that PQQ treatment inhibited myocardial interstitial and perivascular collagen deposition in diabetic mice. Moreover, TGF-*β*1 is a classic pro-fibrotic factor, and activation of the TGF-*β*1/Smad2/3 signaling pathway promotes collagen production. Ma et al. showed that PQQ attenuates the protein expression of TGF-*β*1 in skeletal muscles after sciatic nerve transection [30]. We also found that 40 mg/kg PQQ significantly inhibited the expression of TGF-*β*1 mRNA and protein.

Emerging evidence suggests that diabetes-induced inflammation has a crucial role in the pathogenesis of DCM [31]. The expression and activation of pro-inflammatory cytokines, such as IL-1*β*, IL-6, IL-8, and TNF-*α* are involved in myocardial fibrosis, myocardial hypertrophy, oxidative stress-related injury, and cardiac dysfunction [32]. NF-*κ*B participates in the expression of inflammatory factors as a transcription factor. The NLRP3 inflammasome is a newly identified marker of inflammation in DCM, and NF-*κ*B is able to trigger NLRP3 inflammasome assembly [33]. Meanwhile, HG causes the migration of immune cells into the myocardium and increases macrophage pro-inflammatory M1 polarization, which lead to a marked increase in the levels of TNF-*α*, IL-1*β*, IL-6, and IL-18 [7]. Wilson et al. found that PQQ attenuates the levels of IL-6 and TNF-*α*, and reduces the activation of the MAPK signaling pathway in IL-1*β*-stimulated human synovial sarcoma SW982 cells [34]. Here, we found that PQQ exerted anti-inflammatory effects in DCM, and that PQQ decreased the activation of NF-*κ*B and I*κ*B.

In diabetes, mitochondrial dysfunction results in the over-production of ROS and oxidative stress-related injury, which can directly stimulate NF-*κ*B activation. We found that PQQ increased SOD, GSH-px, and CAT activity; decreased LDH and CK-MB activity; and decreased MDA concentrations. These results indicated that PQQ may be a suitable antioxidant to protect against DCM. Mitochondria are the center of cell metabolism and, as such, are strongly linked to impaired metabolism associated with DCM. Diabetes can cause metabolic abnormalities in body. Hyperglycemia will lead to excessive electron leakage from the electron transport chain to form superoxide ions, causing the mitochondria to produce numerous ROS. ROS aggravates oxidative stress in cells and damages the mitochondrial and cytoplasmic environment. Furthermore, mitochondrial dysfunction will produce more ROS and activate a series of pro-inflammatory pathways that will eventually develop into DCM. Wang et al. [35] reported that swollen mitochondria and reduction in mitochondria count were observed in DCM hearts. Wu’s [15] study showed that early PQQ treatment prevented morphological changes in the heart mitochondria, reduced mitochondrial biogenesis dysfunction caused by pressure overload both in vivo and in vitro and helped maintain heart mitochondrial function. In the present study, we found that 10 nmol/L PQQ can effectively inhibit ROS generation, improve the decrease of mitochondrial membrane potential and maintain mitochondrial function in HG-treated AC16 cells. These results indicate that PQQ may improve DCM by ameliorating mitochondrial damage and reducing ROS production.

Previous studies have reported effects of PQQ on AC16 cells at different concentrations and on other cell types. Wen et al. found that PQQ had no effect on AC16 cells at concentrations from 1 to 200 μmol/L [10]. In our study, although PQQ had no statistically significant effect on cell viability at concentrations from 1 to 10,000 nmol/L, it had adverse effects on AC16 cells at 100,000 nmol/L. Wang et al. showed that PQQ had positive effects on HK-2 cells at 10–10,000 nmol/L, but markedly decreased cell viability after treatment for 48 h at 100,000 nmol/L, alone or in the presence of high glucose concentrations [27]. These results agree with those of our study. We chose 1 and 10 nmol/L to confirm the effect of PQQ on AC16 cells under HG conditions. PQQ was found to decrease pyroptosis-associated protein levels at 10 nmol/L under HG conditions.

Pyroptosis is a type of programmed cell death characterized by an inflammatory response and activation of the NLRP3 inflammasome. The NLRP3 inflammasome is a protein complex, including NLRP3, caspase-1, and ASC, that assembles in response to bacteria, pathogens, and diseases [17, 36]. Assembly of this inflammasome triggers activation of caspase-1 and the maturation of IL-1*β* and IL-18, leading to cell swelling, cell membrane rupture, and the release of cellular contents and inflammatory cytokines [37]. In diabetes, the activation of NF-*κ*B stimulates the transcription of NLRP3 and activation of the NLRP3 inflammasome, resulting in the cleavage of caspase-1, which increases the production of mature IL-1*β* and IL-18 [38]. Recent evidence indicates that silencing the NLRP3 gene and inhibiting the activation of the NLRP3 inflammasome has protective effects against DCM development [39]. Long non-coding RNAs (lncRNAs) are important regulators of pyroptosis. Yang et al. reported that silencing lncRNA-Kcnq1ot1 decreased NLRP3 and caspase-1 expression levels to attenuate myocardial damage in DCM [18]. We found that PQQ treatment ameliorated the activation of the NLRP3 inflammasome signaling pathway, which is a potential mechanism for the cardioprotective role of PQQ.

In conclusion, the results of this study showed that PQQ reduced blood glucose concentrations and ameliorated myocardial hypertrophy and myocardial fibrosis in a model of diabetes. The NF-*κ*B/NLRP3 inflammasome signaling pathway is a key target of PQQ treatment. These findings confirm the in vivo and in vitro benefits of PQQ, a natural nutrient, in the prevention and treatment of DCM. PQQ serves as an important oxidoreductase prosthetic group for redox reaction in vivo and assists quinolase to complete specific biological functions, thereby protecting cells from oxidative stress damage. Supplementing PQQ has a protective effect on many diseases, such as diabetes, osteoporosis and so on [8]. In Japan, Takaoki Kasahara et al. proposed to define PQQ as a new type of vitamin in 2003 [11]; in the United States, FDA included PQQ as a new food ingredient and obtained GRAS certification [40]; in the European Union, the European Food Safety Authority approved PQQ as a new food ingredient [41]. We clarify the role and mechanism of PQQ as a bioactive substance in alleviating the inflammatory death of cardiomyocytes and delaying the development of DCM, providing new ideas for the treatment of DCM. This research will contribute to the application of PQQ in the development of functional foods and new drugs.

## Data Availability

The datasets used and/or analyzed during the current study are available from the corresponding author on reasonable request.

## Abbreviations

ANP: Atrial natriuretic peptide
BNP: Brain natriuretic peptide
CAT: Catalase
CK-MB: Creatine kinase MB
Col I: Collagen I
Col III: Collagen III
CTGF: Connective tissue growth factor
Ctl: Controls
DCM: Diabetic cardiomyopathy
DCM + PQQH: Diabetic mice treated with a high dose of PQQ
DCM + PQQL: Diabetic mice treated with a low dose of PQQ
DCM + PQQM: Diabetic mice treated with a medium dose of PQQ
GSH-px: Glutathione peroxidase
HG: High glucose
IKB: Inhibitor of NF-*κ*B
LDH: Lactate dehydrogenase
MDA: Malondialdehyde
NF-*κ*B: Nuclear factor kappa-B
NLRP3: Nod-like receptor protein 3
PQQ: Pyrroloquinoline quinone
ROS: Reactive oxygen species
SOD: Superoxide dismutase
STZ: Streptozotocin
TGF-*β*1: Transforming growth factor-*β*1
TNF-*α*: Tumor necrosis factor-*α*
*β*-MHC: Myosin heavy chain beta
